# Effectiveness of anlotinib in patients with small‐cell lung cancer and pleural effusion: Subgroup analysis from a randomized, multicenter, phase II study

**DOI:** 10.1111/1759-7714.14176

**Published:** 2021-10-01

**Authors:** Ying Liu, Ying Cheng, Qiming Wang, Kai Li, Jianhua Shi, Lin Wu, Baohui Han, Gongyan Chen, Jianxing He, Jie Wang, Haifeng Qin, Xiaoling Li

**Affiliations:** ^1^ Department of Thoracic Medical Oncology Jilin Cancer Hospital Changchun China; ^2^ Department of Internal Medicine Affiliated Cancer Hospital of Zhengzhou University, Henan Cancer Hospital Zhengzhou China; ^3^ Department of Pulmonary Oncology Tianjin Medical University Cancer Institute and Hospital Tianjin China; ^4^ Department of Medical Oncology Shandong Linyi Tumor Hospital Linyi China; ^5^ Department of Thoracic Medical Oncology The Affiliated Cancer Hospital of Xiangya School of Medicine, Central South University (Hunan Cancer Hospital) Changsha China; ^6^ Department of Respiratory Medicine Shanghai Chest Hospital, Shanghai Jiaotong University Shanghai China; ^7^ Department of Respiratory Medicine Harbin Medical University Cancer Hospital Harbin China; ^8^ Department of Thoracic Surgery The First Affiliated Hospital of Guangzhou Medical University Guangzhou China; ^9^ Department of Thoracic Medical Oncology Cancer Hospital Chinese Academy of Medical Sciences Beijing China; ^10^ Department of Pulmonary Oncology The Fifth Medical Centre of Chinese PLA General Hospital Beijing China; ^11^ Department of Medical Oncology Liaoning Cancer Hospital Shenyang China

**Keywords:** anlotinib, objective response, pleural effusion, small‐cell lung cancer, survival

## Abstract

**Background:**

The presence of pleural effusion is an independent predictor for poor survival in patients with small‐cell lung cancer (SCLC). The aim of this study was to assess the efficacy and safety of anlotinib in patients with SCLC and pleural effusion.

**Methods:**

This was a randomized, double‐blind, multicenter, phase II trial. Patients histologically diagnosed with SCLC and pleural effusion and had received at least two lines of chemotherapy were enrolled into the study. The patients received anlotinib 12 mg/day or a placebo.

**Results:**

The overall response rate (ORR) was 3.7% for anlotinib (*n* = 27) and 0% in the placebo group (*n* = 15) (*p* = 1.000). The disease control rate (DCR) of the anlotinib group (63.0%) was higher than that of the placebo group (0%, *p* < 0.0001). The median progression‐free survival (PFS) increased in the anlotinib group (2.8 months) compared to the placebo group (0.7 months, HR = 0.10, 95% CI: 0.03–0.28, *p* < 0.001). The median overall survival of the anlotinib group (6.5 months) was higher than that of the placebo group (2.8 months, HR = 0.52, 95% CI: 0.22–1.23, *p* = 0.1285). The incidence of any grade adverse events was 100% in both groups. The percentage of grade 3–4 adverse events in the anlotinib group was 44.4% (12/27) compared to 40.0% (6/15) in the placebo group. Hypertension (37.0%), fatigue (29.6%), and loss of appetite (29.6%) typically appeared in the anlotinib group.

**Conclusions:**

In this post hoc analysis, anlotinib was associated with improved PFS in patients with SCLC and baseline pleural effusion. However, additional studies with a large sample size are necessary to substantiate the current findings.

## Graphical abstract

Patients with pleural effusion in this study came from a double‐blind, randomized, multicenter, controlled, phase II study, which was designed to examine the efficacy and safety of anlotinib in limited‐ or extensive‐stage SCLC. The median PFS (mPFS) was higher in the anlotinib group (2.8, 95% CI: 1.4–4.1) months compared to the placebo group (0.7, 95% CI: 0.5–0.7) months. The HR of PFS for the anlotinib group vs. the placebo group was 0.10 (95% CI: 0.03–0.28, *p* < 0.001).

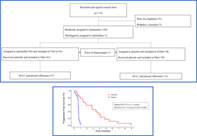

## INTRODUCTION

Lung cancer is the leading cause of cancer‐related mortality; non‐small cell lung cancer (NSCLC) accounts for 85%, and small‐cell lung cancer (SCLC) constitutes the remaining 15% of the lung cancer cases.^1^ Pleural effusion is a common occurrence in patients with SCLC. The seventh edition of IASLC provided a precise classification of the stages of patients with SCLC in 1989, while SCLC with pleural effusion was defined as stage M1.^2^, ^3^ In addition, malignant pleural effusion (MPE) was detected in 11%,^4^ and minimal pleural effusion was present in 20% of patients with SCLC.^5^ The presence of MPE and minimal pleural effusion might be associated with SCLC prognosis.^4^, ^5^

Over the past few decades, novel therapies for NSCLC and SCLC, including chemotherapy, immunotherapy, and targeted drugs, have undergone early exploration.^6^, ^7^, ^8^ First‐line chemotherapy has been found to be effective for MPE, with an efficacy of 74%,^9^ which declines with the advancing lines of treatment.^10^ Intriguingly, both immunotherapy and targeted drugs are limited to provide evidence of the reduced level of MPE in SCLC patients.^11^ Angiogenesis is crucial for the development of MPE. Antiangiogenic agents in combination with chemotherapy have been shown to improve the outcomes of SCLC patients with MPE.^12^ However, the efficacy of these antiangiogenic agents alone in SCLC patients with MPE has not yet been reported.^11^

Angiogenesis plays a critical role in lung cancer development and is considered a hallmark of MPE.^12^, ^13^ Multitargeted tyrosine kinase inhibitors (TKIs) demonstrate significant antitumor effects via inhibition of angiogenic and proliferative signaling.^14^, ^15^, ^16^ Anlotinib is a multitargeted receptor TKI involved in tumor progression. It also inhibits vascular endothelial growth factor (VEGF) isoforms and their receptors (VEGFRs), the platelet‐derived growth factor b (PDGFRb), and the stem cell factor receptor (c‐Kit). Thus, anlotinib could be deemed resistant to tumor cells and angiogenesis.^17^ The ALTER 1202 phase II trial examined the antitumor response of anlotinib in patients with SCLC who failed at least two treatment lines; the drug prolonged the survival of these patients with a tolerable safety profile.^18^, ^19^

To ensure whether anlotinib has a similar antitumor effect in SCLC patients with MPE, a subgroup analysis of MPE patients was performed in this study.

## METHODS

### Patients and study design

This double‐blind, randomized, multicenter, controlled, phase II study was designed to examine the efficacy and safety of anlotinib in limited‐ or extensive‐stage SCLC. The eligible patients were randomized as 2:1 to receive anlotinib or placebo, stratified according to the stage (limited vs. extensive) and pattern of relapse from chemotherapy (sensitive vs. refractory).

Permuted block randomization (*n* = 6/block) was applied within each stratification. Randomization was conducted centrally using the interactive web response system (IWRS) provided by the Department of Biostatistics, School of Public Health of Nanjing Medical University (Nanjing, China). All patients and study personnel were blinded to treatment allocation. The patients received anlotinib 12 mg/day for 14 days every 3 weeks or a placebo. Dose reduction to 10 or 8 mg/day was permitted in cases of toxicity. The treatment was continued until disease progression, the occurrence of intolerable toxicity, physician's decision, or patient's request. However, the crossover was not allowed.

The study was performed in accordance with the Declaration of Helsinki and Guidelines for Good Clinical Practice, as well as laws and regulations in China. The study protocol was approved by the institutional review board in each participating institution. All patients provided written informed consent before any study‐related procedure.

### Eligibility criteria

The eligibility criteria for the original trial were as follows^19^: (1) age 18–75 years; (2) SCLC confirmed by histology; (3) failed at least two lines of chemotherapy; (4) Eastern Cooperative Oncology Group (ECOG) performance status of 0–2; (5) estimated survival >3 months; (6) at least one measurable lesion according to the Response Evaluation Criteria In Solid Tumors version 1.1 (RECIST 1.1); (7) adequate major organ function within 7 days before enrollment.

Patients with central nervous system metastases and/or spinal cord compression were eligible if they were asymptomatic from those conditions or were adequately treated and were stable. The MPE was confirmed by imaging at baseline. Patients with poorly controlled pleural effusion requiring repeated drainage were excluded in the original trial.

### Outcomes and assessments

The primary endpoint was progression‐free survival (PFS, defined as the time from randomization to disease progression according to RECIST 1.1 or death due to any cause, whichever occurred first). The secondary endpoints included overall survival (OS, defined as the time from randomization to death), objective response rate (ORR), disease control rate (DCR), and safety.

Tumor assessments were performed according to RECIST 1.1. The chest, abdominal, and pelvic computed tomography (CT)/magnetic resonance imaging (MRI) were included in the baseline evaluation. The efficacy was evaluated preliminarily after 3 weeks of treatment and confirmed at week 6, followed by two cycles until disease progression was confirmed. Adverse events (AEs) and toxicities were graded according to the National Cancer Institute Common Terminology Criteria for Adverse Events (version 4.03).

The most common AE, hypertension, observed during the trial, was controlled by dose reduction or symptomatic treatments, according to NCI CTCAE 4.03. In the current study, all patients in the anlotinib group began treatment with 12 mg per day. A dose reduction to 10 or 8 mg/day was established as a method to recover from AEs, while no dose adjustment was made in the placebo group.

### Statistical analyses

Continuous data were assessed using the Kolmogorov–Smirnov test for normal distribution. Non‐normally distributed continuous data, such as age and time from diagnosis, are expressed as medians (range/minimum, maximum), while categorical data (sex and ECOG performance status) are expressed as n (%). All statistical analyses were carried out using SAS 9.4. The PFS and OS were estimated by the Kaplan–Meier method and compared between the two groups using the log‐rank test. Hazard ratios (HRs) and 95% confidence intervals (CIs) for PFS and OS were estimated using the Cox proportional hazard model.

The ORR and DCR were compared between the two groups using Pearson's chi‐square test or Fisher's exact test, as appropriate. The ORR DCR confidence intervals were calculated using the exact method based on the binomial distribution (Clopper‐Pearson method). All statistical tests were two‐sided, and *p* < 0.05 was considered statistically significant.

PFS, OS, ORR, and DCR were assessed in the full analysis set (FAS), which included all subjects who received at least one dose of the study drug according to the principle of intention‐to‐treat (ITT). The safety analyses were carried out in the safety analysis set (SS), which included all enrolled patients who received at least one dose of the study drug and had safety records.

## RESULTS

### Patient characteristics

A total of 42 patients with pleural effusion received treatment with a study drug: 27 with anlotinib and 15 with placebo. The baseline characteristics of the patient are presented in Table 1. The median age of the patients in the anlotinib group was 60 (31–70) years. The cohort consisted of 70.4% males, 92.6% were ECOG 0–1, 59.3% had a history or current of smoking. The median age of the patients in the control group was 59 (43–75) years, 73.3% were males, 86.7% were ECOG 0–1, 73.3% had a history of smoking. All patients were stage IV in this study. There were no differences in patient characteristics between the two groups (all *p* > 0.05) (Table 1).

**TABLE 1:** Baseline characteristics of the patients

Variable	Anlotinib (*n* = 27)		Placebo (*n* = 15)		*p*‐value
	*n*	*%*	*n*	*%*	
Age (years)	60 (31–70)		59 (43–75)		0.379
Sex					0.839
Male	19	70.4	11	73.3	
Female	8	29.6	4	26.7	
ECOG performance status					0.632
0	1	3.7	0	0	
1	24	88.9	13	86.7	
2	2	7.4	2	13.3	
Smoking history					0.453
Never	11	40.7	4	26.7	
Former	15	55.6	11	73.3	
Current	1	3.7	0	0	
Previous lines of chemotherapy					0.666
2	22	81.5	13	86.7	
≥3	5	18.5	2	13.3	
Pattern of relapse from chemotherapy^a^					0.219
Sensitive	7	25.9	6	40.0	
Refractory/resistant	20	74.1	8	53.3	
NA	0	0	1	6.7	
Previous radiotherapy					0.654
No	9	33.3	4	26.7	
Yes	18	66.7	11	73.3	

Abbreviations: CR, complete response; ECOG, Eastern Cooperative Oncology Group; NA, not available; PR, partial response.

^a^ Sensitive: first‐line treatment relapse >3 months, refractory/resistant: first‐line relapse ≤3 months.

### Efficacy

As shown in Table 2, among the 27 patients in the anlotinib group, one (3.7%) patient had partial remission (PR), 16 (59.3%) maintained stable disease (SD), and seven (25.9%) patients had progressive disease (PD). In addition, the ORR of the anlotinib and placebo groups was 3.7% and 0%, respectively (*p* = 1.000). The DCR of the anlotinib group (63.0%) was significantly higher than that of the placebo group (0%, *p* < 0.0001). However, no complete response was observed in either of the groups. During the intervention, of the 27 patients in the anlotinib group, three had a significant reduction in MPE. Among the 15 patients in the control group, no significant reduction in pleural effusion was found.

**TABLE 2:** Tumor response

Treatment outcome	Anlotinib (*n* = 27)	Placebo (*n* = 15)	*p*‐value
PR, n (%)	1 (3.7)	0	
SD, n (%)	16 (59.3)	0	
PD, n (%)	7 (25.9)	9 (60.0)	
NE, n (%)	3 (11.1)	6 (40.0)	
ORR (CR + PR), n (%)	1 (3.7)	0	1.000
95% CI	0–19.0	‐	
DCR (CR + PR + SD), n (%)	17 (63.0)	0	<0.0001
95%CI	42.4–80.6	‐	

Abbreviations: CI, confidence interval; DCR, disease control rate; NE, nonevaluable; ORR, objective response rate; PD, progressive disease; PR, partial response; SD, stable disease.

**FIGURE 1:**
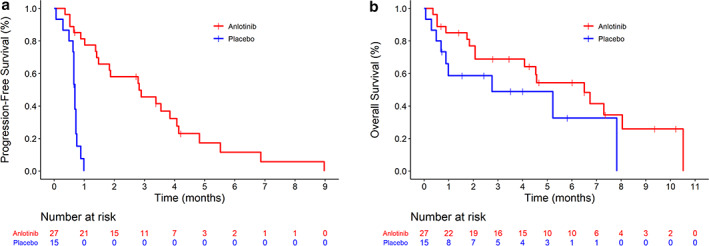
Progression‐free survival (PFS) (a). Overall survival (OS) (b)

The median PFS (mPFS) was higher in the anlotinib group (2.8, 95% CI: 1.4–4.1) months compared to the placebo group (0.7, 95% CI: 0.5–0.7) months. To test the impact of baseline characteristics on PFS, a Cox model was established, and the results showed that the HR of PFS for the anlotinib group versus the placebo group was 0.10 (95% CI: 0.03–0.28, *p* < 0.001) (Figure 1(a)). The mOS of the anlotinib group (6.5, 95% CI: 2.1–8.1) months was 3.7 months higher than that of the placebo group (2.8, 95% CI: 0.5–7.8) months. The HR of OS between the anlotinib and placebo groups was 0.52 (CI: 0.22–1.23, *p* = 0.1285) (Figure 1(b)).

### Safety

The incidence of AEs with anlotinib was higher than that of placebo during any grade. The most common AEs in the anlotinib group were hypertension (37.0%), fatigue (29.6%), and loss of appetite (29.6%), while those in the placebo group were γ‐glutamyl transferase elevation (20.0%), loss of appetite (20.0%), and anorexia (20.0%) (Table 3). The percentage of treatment‐related grade 3 or 4 AEs in the anlotinib group was 44.4% (12/27) compared to 40.0% (6/15) in the placebo group.

**TABLE 3:** AEs that occurred in at least ≥15% of the patients

Adverse events	Anlotinib (*n* = 27)		Placebo (*n* = 15)	
	All grades	Grade 3–4	All grade	Grade 3–4
	n (%)	n (%)	n (%)	n (%)
Any AEs	27 (100.0)	12 (44.4)	15 (100.0)	6 (40.0)
Hypertension	10 (37.0)	2 (7.4)	1 (6.7)	1 (6.7)
Fatigue	8 (29.6)	0	0	0
Loss of appetite	8 (29.6)	0	3 (20.0)	0
Anorexia	8 (29.6)	0	3 (20.0)	0
QT interval prolongation	6 (22.2)	0	2 (13.3)	1 (6.7)
Hand foot syndromes	6 (22.2)	0	0	0
Diarrhea	5 (18.5)	0	0	0
Blood thyroid stimulating hormone increased	5 (18.5)	0	0	0
γ‐glutamyltransferase increased	3 (11.1)	1 (3.7)	3 (20.0)	1 (6.7)

*Note*: QT interval, the time between the start of the Q wave and the end of the T wave in the heart's electrical cycle, when measured using an electrocardiogram.

Abbreviations: AEs, adverse events.

## DISCUSSION

The present study evaluated the efficacy and safety of anlotinib in patients with SCLC and pleural effusion. The results showed that the ORR, DCR, and PFS were higher in the anlotinib group compared to the controls. The OS of the anlotinib group was prolonged by 3.7 months compared to the placebo group but not significantly. Nonetheless, the HR for the PFS was 0.10 in the anlotinib group. The DCR with anlotinib (63.0%) was higher than that with placebo (0%). The occurrence of grade 3–4 AEs was similar in the two groups. Thus, the results indicated that anlotinib is effective in patients with pleural effusion.

Pleural effusion occurs in approximately 11%–20% of patients with SCLC.^4^ Although it can be managed using different treatments, the presence of MPE is associated with short OS. This might be related to poor ECOG and poor efficacy of the existing treatments. The MPE could be reduced by first‐line chemotherapy,^9^ but chemotherapy failed to display a significant improvement in the past decade. Moreover, there is no evidence to prove that immunotherapy is effective in MPE in patients with SCLC.^11^

Angiogenesis played a critical role in the metastatic mechanism of SCLC. Compared to NSCLC, a large number of microvessels are present in SCLC tissues, and antiangiogenic drugs produce a marked effect.^20^, ^21^ Phase II clinical trials showed that the combination of the antiangiogenic drug bevacizumab with chemotherapy demonstrated efficacy and safety in MPE of patients with NSCLC.^22^ NSCLC patients that underwent treatment for MPEs also benefitted from the reduced pleural fluid level; subsequently, alleviated the symptom of dyspnea were alleviated with an overall efficacy OE (CR + IR) rate of 78.6% due to paclitaxel and avastin combination treatment.^23^ In a trial of recurrence of nonsquamous NSCLC patients with unsuccessfully controlled‐MPE, after treatment with bevacizumab plus chemotherapy, pleural effusion control rate (PECR) was 80%. In addition, the median pleural PFS (PPFS) and the median OS reached 16.6 and 19.6 months, respectively.^24^ Thus, the antiangiogenic drugs might have a therapeutic effect on MPE in patients with SCLC. A clinical trial showed that bevacizumab combined with chemotherapy is effective in patients with SCLC,^25^ but only limited data have proved its efficacy in patients with SCLC combined with MPE.

In this study, the PFS of the anlotinib group was significantly longer than that of the placebo group. A higher DCR was involved in the prolongation of PFS, which could be attributed to the antitumor effect of anlotinib in patients with SCLC.^18^ The FAS analysis of the 1202 trial showed that the DCR and PFS had a superior value in the anlotinib group, which was similar in patients with MPE. However, the great improvement of anlotinib on the PFS and OS in this subgroup suggested a strong anticancer effect on MPE. Yet, the PFS and OS of patients with MPE were slightly lower than in the FAS population in the previous study. This phenomenon was similar to that of the SCLC chemotherapy study, indicating that MPE might be an adverse factor in the prognosis of antiangiogenic TKI treatment.

In this study, patients with MPE were studied because of their poor prognosis, but the improvement in prognosis was not necessarily due to the MPE alleviation alone since anlotinib is a systemic therapy. Additional studies are necessary to determine the clinical impact of directly managing the MPE. In this sense, future studies should specifically examine the combination of anlotinib with local therapies in patients with MPE. Such therapies include pleurodesis, localized immunotherapy, the therapeutic use of pleural‐infiltrating T cells, and immune checkpoint inhibitors.^26^, ^27^

The toxicities were tolerable in this study. The AE profile of the anlotinib group was similar to that in the original trial.^19^ The most common AE in the anlotinib group was hypertension (37.0%), and no grade 5 AEs were observed in this study. The difference in the occurrence of grade 3–4 AEs (4.4%) was probably not clinically significant, but the sample size of the present subgroup analysis was too small to reach firm conclusions in this regard. It will have to be examined more closely in future trials. Notwithstanding this, the improvement in OS, PFS, and DCR could justify the use of anlotinib as a palliative approach, especially given that the rates of grade 3–4 AEs are not greatly increased. In the original trial of 81 patients on anlotinib and 39 on placebo, the difference in grade 3–4 AEs was 8.3%, with an improvement of 3.4 months in PFS and 2.4 months in OS.^19^

Nevertheless, the present study has several limitations. A small number of patients were enrolled, and hence the data were not supported by strong evidence. Although the OS tended to improve, it lacked statistical difference, thereby necessitating additional studies.

In conclusion, the presence of MPE in patients with SCLC is common. It has limited therapeutic methods, especially in the third‐line and later treatment options. Anlotinib is effective and tolerable, providing a novel treatment option for patients with SCLC and MPE.

## CONFLICT OF INTEREST

All authors declare there are no conflicts of interest.
